# An Update on the Emerging Role of Resistin on the Pathogenesis of Osteoarthritis

**DOI:** 10.1155/2019/1532164

**Published:** 2019-01-28

**Authors:** Cheng-Wu Zhao, Yu-Hang Gao, Wen-Xia Song, Bo Liu, Lu Ding, Ning Dong, Xin Qi

**Affiliations:** ^1^Department of Sports Medicine, The First Hospital of Jilin University, Changchun, Jilin 130021, China; ^2^Department of Orthopaedic Surgery, The First Hospital of Jilin University, Changchun, Jilin 130021, China; ^3^Department of Pathology, The First Hospital of Jilin University, Changchun, Jilin 130021, China

## Abstract

**Background:**

Resistin may be involved in the pathogenesis of osteoarthritis (OA), but a systematic understanding of the role of resistin in OA is lacking.

**Methods:**

We reviewed studies that evaluated the role of resistin in OA. The expression levels of resistin in vitro experiments and OA/rheumatoid arthritis (RA) patients were analyzed. We also studied potential resistin receptors and the signaling pathways that these receptors activate, ultimately leading to cartilage degeneration.

**Results:**

Resistin levels in both the serum and synovial fluid were higher in OA and RA patients than in healthy subjects. Overall, resistin levels are much higher in serum than in synovial fluid. In human cartilage, resistin induces the expression of proinflammatory factors such as degradative enzymes, leading to the inhibition of cartilage matrix synthesis, perhaps by binding to Toll-like receptor 4 and the adenylyl cyclase-associated protein 1 receptor, which then activates the p38-mitogen-activated phosphate kinase, protein kinase A–cyclic AMP, nuclear factor-*κ*B, and C/enhancer-binding protein *β* signaling pathways.

**Conclusion:**

Resistin levels are higher in OA patients than in healthy controls; however, the precise role of resistin in the pathogenesis of OA needs to be studied further. Resistin may be a novel therapeutic target in OA in the future.

## 1. Introduction

Osteoarthritis (OA) is characterized by the progressive deterioration of the articular cartilage and structural changes to the entire synovial joint, including the synovium [1], meniscus of knee [2], adipose tissue [3], periarticular ligaments [4], and subchondral bone [5]. Cartilage degeneration is a hallmark of the process of OA [6]. Chondrocytes, present in the adult articular cartilage, are the only cell population surrounded by the extracellular matrix (ECM) [7]. As cartilage degeneration continues, the loss of ECM and disturbance of cartilage homeostasis lead to the propagation of chondrocyte death and tissue degeneration [8]. The pathophysiological mechanism of OA generally involves subchondral bone remodeling, osteophyte formation, synovial inflammation, ligamentous laxity, and the weakening of periarticular muscles. However, the trigger of OA is not clearly understood because of its diverse etiological factors, which may be attributable to interactions between systemic and local factors [9]. Numerous risk factors for OA have been identified. Systemic risk factors include old age, female gender, overweight and obesity, ethnicity, and genetic variables, while local risk factors include previous knee injury, repetitive use of joints, bone density, muscle weakness, and joint laxity [10]. While obesity has been implicated in OA, the underlying pathology may not simply be due to the increased mechanical stresses, but rather it may involve soluble factors such as adipokines [11]. Adipokines are a family of cytokines secreted primarily by adipose tissue and associated with cartilage degeneration and synovial inflammation in OA [12]. Resistin, an adipokine that has been observed in synovial fluid (SF) and synovial tissues from OA patients, has been shown to contribute to OA progression [13].

Resistin is a 12 kDa cysteine-rich polypeptide hormone protein secreted by macrophages and adipocytes in humans and mice, respectively. It is the founding member of the resistin-like molecule (RELM) hormone family, and it consists of 108 amino acid peptides; in human blood, it circulates as a dimeric protein consisting of two 92-amino acid polypeptides [14, 15]. It is produced by white and brown adipose tissues, but it also has been identified in several other peripheral tissues. The site of resistin production is species dependent. In mice, resistin is produced by adipocytes, whereas in humans, it is predominantly expressed in macrophages [16]. Murine resistin is mainly implicated in the pathogenesis of obesity-mediated insulin resistance and type 2 diabetes, but the concrete target cells remain inconclusive [17, 18]. However, some authors believe that in humans, immune cells are found to be recruited by resistin and participate in inflammatory processes [19–21].

Resistin suppresses the ability of insulin to stimulate cellular glucose uptake and plays a role in obesity, insulin resistance, diabetes [22], rheumatoid arthritis (RA), and OA [23, 24]. Some authors believe that in humans, resistin plays a more important role in inflammatory processes than in insulin resistance, as serum resistin levels correlate better with subclinical inflammation than with insulin resistance [25]. In addition, it has been proven in studies that human resistin alone can promote inflammation [19, 20, 26, 27], while other studies have also shown that human resistin may exert anti-inflammation in response to a fatal endotoxin challenge [28]. These conflicting findings point towards the idea that the proinflammatory or anti-inflammatory function of resistin is context related and disease specific. The general consensus is that resistin in metabolism plays a pathologic role in promoting insulin resistance, atherosclerosis, and hypertension, whether in mouse or human studies [29–31]. In humans, resistin levels have been positively associated with central/visceral obesity (but not BMI) and play a role in proinflammatory processes [32]. In healthy subjects, resistin expression was also detected. The findings from a cross-sectional study of 6636 adults recruited randomly indicated that mean resistin level was slightly higher in women (6.06 ± 2.41 ng/mL vs. 5.63 ± 2.18 ng/mL) for these healthy people [33].

Recently, some authors have suggested that resistin is expressed in the synovial joints of RA and OA patients [34, 35]. Resistin upregulates the expression of inflammatory cytokines and chemokines in chondrocytes, and intra-articular resistin injection induces arthritis in healthy mouse joints [20]. Other studies have shown that serum resistin levels are higher in patients with severe OA than in controls without OA, and that resistin is present in both serum and SF, suggesting its systemic and local involvement in the inflammatory changes of OA [13]. The role of resistin in OA could be explained by its action as an inducer of proinflammatory cytokines, such as tumor necrosis factor- (TNF-) *α*, interleukin- (IL-) 6, and IL-12, via the nuclear factor- (NF-) *κ*B signaling pathway in diverse inflammatory conditions [36].

The current studies mostly support the notion that human resistin is highly secreted and exerts proinflammatory actions in OA. Thus far, there is only a recent meta-analysis published in 2014, which reported that high resistin expression represents a significant and reproducible marker of rapid disease progression in OA patients, especially male patients [37]. They just summarize resistin expression linked with OA; however, the role of resistin in the pathogenesis of OA has not yet been systematically elucidated. Therefore, it is necessary to update the diagnostic value and accuracy of human resistin expression and recapitulate its known pathogenesis in OA. In this review, we have attempted to summarize the characteristics of resistin expression in OA and detail the underlying pathogenetic mechanisms via which resistin may contribute to the development of OA.

## 2. Role and Expression of Resistin in OA

### 2.1. Role of Resistin in OA

Resistin has been less widely investigated in OA patients, but it has been associated with radiographic knee OA [38]. Recent epidemiological studies have shown that serum and SF resistin levels are positively correlated with OA severity [39–41]. In patients with hand OA, the circulating levels of resistin positively correlate with leptin levels [36, 42]. Resistin has also been reported to induce the production of matrix metalloproteinase (MMP) and aggrecans in human chondrocytes and to promote the release of proinflammatory cytokines such as IL-6 and TNF-*α* [43]. In clinical studies, serum resistin levels were positively and independently associated with cartilage and bone marrow lesions (BMLs) observed on magnetic resonance imaging in patients with knee OA [44]. However, other authors have found that in knee OA patients, the resistin level in SF but not in serum is significantly associated with Noyes scores, Kellgren-Lawrence grades, and WOMAC pain, physical function, and total scores as well as the level of C-telopeptides of type II collagen [45]. In one study of knee OA patients, the SF resistin level was mainly related to joint dysfunction and only weakly related to joint pain [40]. The serum resistin level was significantly associated with the number of symptomatic joints in men, with higher levels being associated with a lower overall count [13].

The role of resistin in OA remains a matter of controversy. One study of 50 knee OA patients and 50 sex-matched healthy subjects evaluated serum resistin levels, knee OA scores, and lean mass and fat mass data obtained using dual-energy X-ray absorptiometry; the results showed that resistin levels did not differ between patients and controls [46]. Furthermore, resistin is not associated with cartilage volume loss in the global knee [41].

In summary, resistin itself does not damage joints and cartilages, and its effects on cartilage are two tiered. In addition to promoting the synthesis of cartilage proteoglycans, the main role of resistin, as far as we know, is to promote the progression of OA. Thus, resistin levels may be valuable for early prognostication in OA patients.

### 2.2. SF and Serum Resistin Levels in OA Patients

In OA patients, resistin levels were found to be lower in the SF (0.75 ng/mL) than in the plasma (4.1 ng/mL), and the SF resistin level positively correlates with the SF levels of IL-6, MMP-1, and MMP-3 [47]. Another study consecutively enrolled 205 OA patients; they found that the median level of serum resistin was 2.37 ng/mL, and serum levels were positively and independently associated with cartilage defects and BMLs in patients with knee OA [44]. Furthermore, resistin is released from cultured OA cartilage, and the resistin levels in culture correlate with the resistin levels in SF [48]. Finally, neither SF nor serum resistin levels differ between diabetic versus nondiabetic patients or between sexes [42]. In a study of 80 female OA patients (43 with Mets-OA and 37 with non-Mets-OA), the SF resistin levels (4.7 vs. 4.3 ng/mL) and the plasma resistin levels (10.2 vs. 10.9 ng/mL) did not differ between the Mets-OA and non-Mets-OA groups, respectively [49].

During the past few years, many advances have been made in our understanding of the relationship between resistin and obesity. In newborns, the adipokine level has been associated with early childhood growth; higher resistin levels at birth are associated with higher odds of being overweight at 2–3 years of age [50]. Among young individuals (18–30 years; *n* = 47), plasma resistin levels were found to be higher in those who were overweight or obese than in eutrophic subjects [51]. Among adults, serum resistin levels were observed to be significantly higher in obese subjects than in lean subjects, and the circulating levels of resistin were positively correlated with body mass index and fat mass [52].

Some studies have examined resistin expression in males and females. In one study of 35 OA patients, the mean SF resistin level was 7.26 ± 1.21 ng/mL (range, 1.62–26.41 ng/mL), and this level did not significantly differ between female (8.47 ± 1.75 ng/mL) and male patients (5.73 ± 1.58 ng/mL) [35]. Another study found a significant positive correlation between the SF levels of IL-6 and resistin in female subjects but not in male subjects [53].

The data on sex-related differences in serum resistin levels are contradictory. In one study involving patients with late-stage knee OA scheduled for joint replacement surgery, no significant differences were found in serum resistin levels between female (26.6 ng/mL) and male patients (26.1 ng/mL) [13]. Another study reported that serum resistin levels were 2–3 times higher in female patients than in male patients [54]. In contrast to the findings, one study observed that serum resistin levels were higher in males (9.4 ng/mL) than females (7.3 ng/mL) [55]. However, most studies report that both SF and serum resistin levels are higher in females than in males, and very few studies report the reverse.

In a cross-sectional study of 156 female subjects, including 60 patients with nonradiographic hand OA, 50 with radiographic hand OA, and 46 controls, serum resistin levels were higher in the radiographic hand OA group than in the control and nonradiographic hand OA groups, indicating that serum resistin levels are associated with radiographic changes in hand OA, especially for subchondral erosion [36]. Another study enrolled 94 Caucasian outpatients diagnosed with nonerosive (NE) and erosive (E) hand osteoarthritis (HOA). When the two groups (47 NE-HOA patients and 47 E-HOA patients) were compared with normal control groups (21 subjects), significantly higher resistin levels were found in both E-HOA and NE-HOA groups (5.8 pg/mL and 5.6 pg/mL) than control healthy groups (3.7 pg/mL) [56].

Only a few articles have reported that serum resistin levels do not significantly differ among RA and OA patients and those without arthritis (12.1, 10.8, and 10.0 ng/mL, respectively) [57]. Similarly, a single case-control study has also shown that no statistical differences were found between knee OA groups (9.8 ± 0.8 ng/mL, *N* = 50) and healthy subjects (10.7 ± 0.7 ng/mL, *N* = 50) [46].

Overall, the majority of the literature indicates that resistin expression is higher in OA patients than in healthy controls, suggesting that resistin expression is closely related to the development of OA. Thus, resistin may be a new biomarker for the diagnosis of disease severity in OA. More studies are required to confirm this and to determine suitable threshold values for diagnostic purposes.

## 3. Possible Pathogenetic Mechanisms Underlying Resistin-Induced OA

Resistin is the founding member of the RELM protein family, and in human beings, it plays a crucial role in systemic inflammation, especially OA-related inflammation [25, 43, 58]. The chronic inflammatory response in OA results from a failure or an absence of the mechanisms responsible for maintaining homeostasis and the persistence of mechanisms that upregulate inflammation. Articular cartilage degeneration is the key pathological change of OA, whereas the articular cartilage contains only a single type of cell referred to as chondrocytes which reside within an ECM rich in collagen fibers [7, 59]. Thus, chondrocytes are the directed target cells of the resistin-related OA process. The pathway of resistin-induced OA may involve binding to its own receptors in chondrocytes, the activation of some signaling pathways, signal translocation into the cell nucleus, and finally, increased expression of genes related to the cytokines/chemokines that catabolize cartilage. From current knowledge, there are three possible functions of resistin in OA: (i) to induce the expression of inflammatory cytokines and chemokines, which elicit a proinflammatory action; (ii) to enhance the production of tissue-degrading enzymes, which promote cartilage destruction; and (iii) to downregulate structural proteins of the hyaline cartilage, which are critical components of the pericellular matrix [20, 43, 47, 53, 60, 61]. In order to further evaluate how resistin works in resistin-induced OA, the concrete receptors, signaling pathway, and expression of cytokines and chemokines stimulated by resistin are all needed to make a summary.

### 3.1. Resistin Receptors

Resistin was first identified as a mediator that potentially links obesity to diabetes [16]. In rodents, it is secreted by mature adipocytes [16], and in humans, it is primarily secreted by monocytes and macrophages [58] and induces low-grade inflammation. Therefore, resistin has different types of functional receptors in rodents and humans. The binding of resistin to these receptors is the first step in its activation and the subsequent cascade of events that leads to low-grade inflammation.

Four distinct functional receptors that bind resistin have been identified: Toll-like receptor 4 (TLR4), an isoform of decorin, receptor tyrosine kinase-like orphan receptor 1 (ROR1), and adenylyl cyclase-associated protein 1 (CAP1).

#### 3.1.1. TLR4

In 2010, Tarkowski et al. first demonstrated that a cell surface receptor mediates the proinflammatory effects of resistin and regulates carbohydrate metabolism by competing with lipopolysaccharide for binding to TLR4 in human leucocytes, monocytic cell line THP1, and epithelial cells (HEK293) [62]. A 2017 study of intervertebral disc degeneration showed that resistin binds to TLR4 and increases CCL4 expression in nucleus pulposus cells, causing macrophage infiltration [63]. In a neuronal cell study, resistin was found to inhibit neuronal autophagy by binding to TLR4 in wild-type mice but not in *TLR4* gene knockout mice [64]. The resistin-induced activation of porcine alveolar macrophages via TLR4 promotes the production of proinflammatory cytokines [65]. These studies indicate that TLR4 as a cell surface receptor plays an important role in resistin-induced inflammation. However, there has been no evidence of direct binding between TLR4 and human resistin, and so, further biochemical assays are needed, especially in OA.

#### 3.1.2. Decorin

In mice, an isoform of decorin (Δdecorin) was found to act as a functional receptor of resistin on the surface of adipose progenitor cells and to possibly regulate white adipose tissue expansion [66]. In humans, decorin is scarcely expressed in mononuclear and macrophage cells, and one study found that decorin expression levels are not increased after resistin treatment. However, the decorin used in that study was an extracellular cleavage product, and the necessary proteolytic cleavage may not occur in the model cell line employed [67].

#### 3.1.3. ROR1

A 2012 study showed that in mice, ROR1 can mediate some of the functions of resistin in 3T3-L1 adipogenesis and glucose uptake, demonstrating for the first time that ROR1 may be a potential receptor for mouse resistin in 3T3-L1 cells and whose complete ROR1 protein is at the cell plasma membrane [68]. However, ROR1 is scarcely expressed in human mononuclear and macrophage cells, and in human cells, ROR1 expression does not increase after resistin treatment [67].

#### 3.1.4. CAP1

Human resistin directly binds to CAP1 in monocytes and white adipose tissue and upregulates the transcription of inflammatory cytokines; the suppression of CAP1 expression downregulates this resistin-mediated inflammatory activity both in vitro and in vivo [67]. Lee et al. [67] considered that these gain- and loss-of-function studies indicate that CAP1 is a bona fide functional receptor for human resistin. Furthermore, CAP1 was found to be located on the cell membrane of the surface of monocytes. However, it lacks a transmembrane domain; thus, the cell biological mechanism underlying its membrane location is unclear and will require future investigation [67, 69]. Another study found that the expression of resistin and CAP1 in synovial tissue is stronger in RA than in OA, implying that CAP1 contributes to the pathogenesis of RA by increasing chemokine production by fibroblast-like synoviocytes [70].

**Figure 1:**
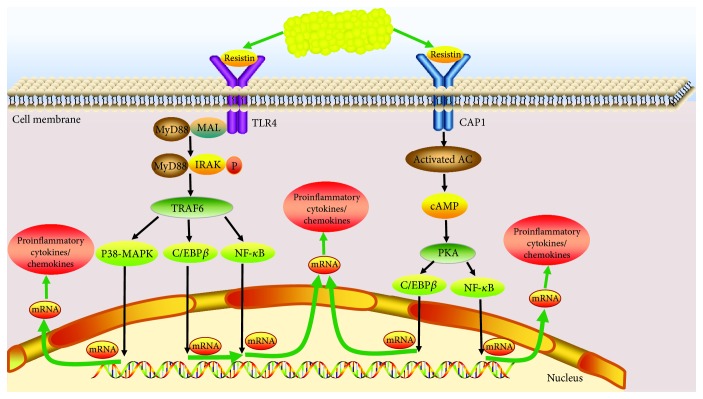
The potential proinflammatory role of resistin in the pathogenesis of osteoarthritis. Resistin possibly binds to the transmembrane Toll-like receptor 4 (TLR4) and activates TNF receptor-associated factor 6 (TRAF6) through the MyD88-dependent signaling pathway, which then activates the p38-mitogen-activated protein kinase (MAPK), C/enhancer-binding protein *β* (EBP*β*), and nuclear factor- (NF-) *κ*B signaling pathways, leading to the activation of the transcription of proinflammatory cytokine genes in the cell nucleus. Resistin can also stimulate the expression of proinflammatory cytokines and chemokines through the p38-MAPK and NF-*κ*B pathways by binding to adenylyl cyclase-associated protein 1 (CAP1) receptors, which activates the cyclic AMP (cAMP)-protein kinase A (PKA) signaling pathway.

Thus, only four resistin receptors have been discovered in mice and humans. Upon reviewing the literature on the role of these four receptors, we conclude that resistin interacts with different receptors depending on the tissue and cell type. Decorin and ROR1 are receptors for murine resistin and do not seem to mediate inflammatory responses in humans. TLR4 is the most studied in humans, but all the studies thus far have merely reported the interactions between human resistin and TLR4 but have not provided the results of direct-binding assays. CAP1 has been proved to directly bind to resistin on the cell membrane of the human monocyte surface and to lead to inflammation; however, no resistin receptor has been identified in chondrocytes. Thus, TLR4 and CAP1 may be the most likely receptors for human resistin in chondrocytes, but further studies are required to confirm this (Figure 1).

### 3.2. Proinflammatory Signaling Pathway of Resistin

Receptors that bind resistin are mostly located in the cytomembrane or cytosol. The resistin-induced activation of these receptors leads to the upregulation of proinflammatory cytokines and chemokines at both the protein and mRNA levels, implying the existence of a signaling pathway connecting resistin-mediated receptor activation and the observed proinflammatory effects. Many studies have reported that the stimulation of human monocytes, macrophages, fibroblast-like synoviocytes, nucleus pulposus cells, and articular chondrocytes by resistin results in the upregulation of proinflammatory cytokines and chemokines [19, 60, 61, 63, 67, 70]. The signaling pathways that have been found to be activated by resistin include the cyclic AMP (cAMP)/protein kinase A- (PKA-) dependent, p38-mitogen-activated phosphate kinase (MAPK), C/enhancer-binding protein *β* (EBP*β*), and NF-*κ*B pathways.

In monocytes and macrophages, the cAMP/PKA-dependent signaling pathway might be the key mechanism via which resistin binding to CAP1 receptors upregulates the intracellular cAMP concentration, PKA activity, and NF-*κ*B-related transcription of many inflammatory cytokines [67]. In nucleus pulposus cells, resistin treatment leads to a rapid increase in the phosphorylation of p38 and NF-*κ*B [63]. In the same cells, resistin was found to upregulate CCL4 expression by activating the p38-MAPK and NF-*κ*B signaling pathways; furthermore, when these pathways were inhibited, CCL4 expression was downregulated [63]. In human chondrocytes, C/EBP*β* gradually and steadily induces the upregulation of the chemokines CCL3 and CCL4 in response to IL-1*β* [71]. Human chondrocytes stimulated with resistin present the same upregulation, which is mediated through the NF-*κ*B signaling pathway [60, 61]. These reports suggest that C/EBP*β* is involved in the resistin-induced upregulation of chemokine genes.

A signaling pathway mediates the transmission of an extracellular molecular signal to the interior of the cell through the cell membrane. The function of resistin can be inferred via the signaling pathway that it activates. In human chondrocytes, resistin has been proven to activate the C/EBP*β* and NF-*κ*B pathways. There is abundant room for further investigation on the signaling pathways mediating the effects of resistin in human chondrocytes.

### 3.3. Expression of Cytokines and Chemokines Stimulated by Resistin in OA

The proinflammatory properties of resistin have been observed in animal models and human tissue samples [20, 43, 47, 61]. Numerous cytokines and chemokines are upregulated by resistin, making it a novel and interesting therapeutic target in chronic inflammatory diseases such as RA and OA. Thus far, the proinflammatory properties of resistin have been studied in human knee fibroblast-like synoviocytes from RA patients, human monocytes/macrophages, and human knee articular chondrocytes.

In human knee fibroblast-like synoviocytes harvested from RA patients and incubated with resistin in vitro, the expression levels of 18 molecules were more than 2-fold higher in resistin-stimulated cells than in unstimulated cells. These upregulated molecules included the seven chemokines, CXCL1, CXCL2, CXCL3, CXCL5, CXCL6, CXCL8, and CCL2, as well as IL-6 [70].

Consistent with the results in fibroblast-like synoviocytes, another study showed that treatment of human primary monocytes with recombinant human resistin (10 *μ*g/mL) for 48 h led to the release of cytokines and chemokines, such as IL-1*α*, IL-1*β*, IL-1R*α*, IL-6, IL-7, CCL4/macrophage inflammatory protein- (MIP-) 1*α*, CCL3/MIP-1*β*, CXCL8/IL-8, IL-10, CCL2/methyl-accepted chemotaxis protein- (MCP-) 1, granulocyte colony-stimulating factor (G-CSF), and TNF-*α* [43]. Other researchers have found that resistin leads to the upregulation of the mRNA and protein levels of inflammatory cytokines such as IL-6, TNF-*α*, and IL-1*β* via the PKA and NF-*κ*B signaling pathways [67].

OA is characterized by cartilage degeneration, soft tissue damage, and inflammation. In OA, chondrocytes are ultimately damaged by proinflammatory chemokines, cytokines, and other molecules. Compared to knee chondrocytes from healthy controls, those from OA patients exhibited a greater than 10-fold increase in the expression of IL-1*α*, IL-1*β*, CCL3, CCL3L1, CCL4, CCL5, CCL8, CCL20, CXCL1, CXCL2, CXCL3, CXCL5, CXCL6, CXCL8 (IL-8), MMP-1, and MMP-13 and a less than 10-fold increase in the expression of TNF*α*, CCL2, CX3CL1, and ADAMTS-4 in response to resistin treatment [60].

These above results further support the notion that resistin plays a proinflammatory role in cell metabolism, and the cytokines/chemokines triggered by resistin play a larger role in promoting catabolism than anabolism in OA.

## 4. Conclusion

OA is a multifactorial disease, and the factors that lead to the degeneration of joint cartilage include mechanical stresses and proinflammatory cytokines/chemokines. The current body of evidence shows that a positive correlation exists between resistin and OA. Resistin upregulates proinflammatory cytokines/chemokines in OA, thereby increasing catabolic activity in cartilage. Thus, resistin is a potential biomarker for the diagnosis of OA. However, the direct receptor for resistin and its corresponding signaling pathways need to be further investigated to elucidate the underlying pathogenetic mechanism in OA. Thus far, very few studies have investigated the interaction of resistin with cytokines/chemokines. Future work to identify the bona fide receptor of resistin, its corresponding signaling pathways, and the cytokines that mediate resistin-induced inflammatory responses is required to conclusively define the role of resistin in OA progression.

## References

[1] Bhattaram P., Chandrasekharan U. (2017). The joint synovium: a critical determinant of articular cartilage fate in inflammatory joint diseases. *Seminars in Cell & Developmental Biology*.

[2] Atik O. S., Erdogan D., Seymen C. M., Bozkurt H. H., Kaplanoglu G. T. (2016). Is there crosstalk between subchondral bone, cartilage, and meniscus in the pathogenesis of osteoarthritis?. *Joint Diseases and Related Surgery*.

[3] Conde J., Scotece M., Lopez V. (2014). Differential expression of adipokines in infrapatellar fat pad (IPFP) and synovium of osteoarthritis patients and healthy individuals. *Annals of the Rheumatic Diseases*.

[4] Hasegawa A., Otsuki S., Pauli C. (2012). Anterior cruciate ligament changes in the human knee joint in aging and osteoarthritis. *Arthritis and Rheumatism*.

[5] Goldring S. R. (2012). Alterations in periarticular bone and cross talk between subchondral bone and articular cartilage in osteoarthritis. *Therapeutic Advances in Musculoskeletal Disease*.

[6] Malemud C. J., Martel-Pelletier J., Pelletier J. P. (1987). Degradation of extracellular matrix in osteoarthritis: 4 fundamental questions. *The Journal of Rheumatology*.

[7] Cucchiarini M., de Girolamo L., Filardo G. (2016). Basic science of osteoarthritis. *Journal of Experimental Orthopaedics*.

[8] Temple M. M., Bae W. C., Chen M. Q. (2007). Age- and site-associated biomechanical weakening of human articular cartilage of the femoral condyle. *Osteoarthritis and Cartilage*.

[9] Heijink A., Gomoll A. H., Madry H. (2012). Biomechanical considerations in the pathogenesis of osteoarthritis of the knee. *Knee Surgery, Sports Traumatology, Arthroscopy*.

[10] Zhang Y., Jordan J. M. (2010). Epidemiology of osteoarthritis. *Clinics in Geriatric Medicine*.

[11] Gabay O., Hall D. J., Berenbaum F., Henrotin Y., Sanchez C. (2008). Osteoarthritis and obesity: experimental models. *Joint, Bone, Spine: Revue du Rhumatisme*.

[12] Bijlsma J. W. J., Berenbaum F., Lafeber F. P. J. G. (2011). Osteoarthritis: an update with relevance for clinical practice. *The Lancet*.

[13] Perruccio A. V., Mahomed N. N., Chandran V., Gandhi R. (2014). Plasma adipokine levels and their association with overall burden of painful joints among individuals with hip and knee osteoarthritis. *The Journal of Rheumatology*.

[14] Tiaka E. K., Manolakis A. C., Kapsoritakis A. N., Potamianos S. P. (2011). The implication of adiponectin and resistin in gastrointestinal diseases. *Cytokine & Growth Factor Reviews*.

[15] Sood A., Shore S. A. (2013). Adiponectin, leptin, and resistin in asthma: basic mechanisms through population studies. *Journal of Allergy*.

[16] Steppan C. M., Bailey S. T., Bhat S. (2001). The hormone resistin links obesity to diabetes. *Nature*.

[17] Nakata M., Okada T., Ozawa K., Yada T. (2007). Resistin induces insulin resistance in pancreatic islets to impair glucose-induced insulin release. *Biochemical and Biophysical Research Communications*.

[18] Li F. P., He J., Li Z. Z., Luo Z. F., Yan L., Li Y. (2009). Effects of resistin expression on glucose metabolism and hepatic insulin resistance. *Endocrine*.

[19] Silswal N., Singh A. K., Aruna B., Mukhopadhyay S., Ghosh S., Ehtesham N. Z. (2005). Human resistin stimulates the pro-inflammatory cytokines TNF-*α* and IL-12 in macrophages by NF-*κ*B-dependent pathway. *Biochemical and Biophysical Research Communications*.

[20] Bokarewa M., Nagaev I., Dahlberg L., Smith U., Tarkowski A. (2005). Resistin, an adipokine with potent proinflammatory properties. *The Journal of Immunology*.

[21] Krysiak R., Handzlik-Orlik G., Okopien B. (2012). The role of adipokines in connective tissue diseases. *European Journal of Nutrition*.

[22] Jamaluddin M. S., Weakley S. M., Yao Q., Chen C. (2012). Resistin: functional roles and therapeutic considerations for cardiovascular disease. *British Journal of Pharmacology*.

[23] Su C. M., Hsu C. J., Tsai C. H., Huang C. Y., Wang S. W., Tang C. H. (2015). Resistin promotes angiogenesis in endothelial progenitor cells through inhibition of microRNA206: potential implications for rheumatoid arthritis. *Stem Cells*.

[24] Boström E. A., Svensson M., Andersson S. (2011). Resistin and insulin/insulin-like growth factor signaling in rheumatoid arthritis. *Arthritis and Rheumatism*.

[25] Senolt L., Housa D., Vernerova Z. (2006). Resistin in rheumatoid arthritis synovial tissue, synovial fluid and serum. *Annals of the Rheumatic Diseases*.

[26] Jiang S., Park D. W., Tadie J. M. (2014). Human resistin promotes neutrophil proinflammatory activation and neutrophil extracellular trap formation and increases severity of acute lung injury. *Journal of Immunology*.

[27] Lago F., Dieguez C., Gomez-Reino J., Gualillo O. (2007). Adipokines as emerging mediators of immune response and inflammation. *Nature Clinical Practice Rheumatology*.

[28] Jang J. C., Li J., Gambini L. (2017). Human resistin protects against endotoxic shock by blocking LPS-TLR4 interaction. *Proceedings of the National Academy of Sciences*.

[29] Jiang Y., Lu L., Hu Y. (2016). Resistin induces hypertension and insulin resistance in mice via a TLR4-dependent pathway. *Scientific Reports*.

[30] Kim S.-J., Nian C., McIntosh C. H. S. (2013). Resistin knockout mice exhibit impaired adipocyte glucose-dependent insulinotropic polypeptide receptor (GIPR) expression. *Diabetes*.

[31] He Y., Bai X. J., Li F. X. (2016). Resistin may be an independent predictor of subclinical atherosclerosis formale smokers. *Biomarkers*.

[32] Zhang J., Qin Y., Zheng X. (2002). The relationship between human serum resistin level and body fat content, plasma glucose as well as blood pressure. *Zhonghua Yi Xue Za Zhi*.

[33] Marcelino-Rodríguez I., Almeida Gonzalez D., Alemán-Sánchez J. J. (2017). Inverse association of resistin with physical activity in the general population. *PLoS One*.

[34] de Boer T. N., van Spil W. E., Huisman A. M. (2012). Serum adipokines in osteoarthritis; comparison with controls and relationship with local parameters of synovial inflammation and cartilage damage. *Osteoarthritis and Cartilage*.

[35] Presle N., Pottie P., Dumond H. (2006). Differential distribution of adipokines between serum and synovial fluid in patients with osteoarthritis. Contribution of joint tissues to their articular production. *Osteoarthritis and Cartilage*.

[36] Choe J. Y., Bae J., Jung H. Y., Park S. H., Lee H. J., Kim S. K. (2012). Serum resistin level is associated with radiographic changes in hand osteoarthritis: cross-sectional study. *Joint, Bone, Spine: Revue du Rhumatisme*.

[37] Li X. C., Tian F., Wang F. (2014). Clinical significance of resistin expression in osteoarthritis: a meta-analysis. *BioMed Research International*.

[38] van Spil W. E., Welsing P. M. J., Kloppenburg M. (2012). Cross-sectional and predictive associations between plasma adipokines and radiographic signs of early-stage knee osteoarthritis: data from CHECK. *Osteoarthritis and Cartilage*.

[39] Zheng S., Xu J., Xu S. (2016). Association between circulating adipokines, radiographic changes, and knee cartilage volume in patients with knee osteoarthritis. *Scandinavian Journal of Rheumatology*.

[40] Calvet J., Orellana C., Albinana Gimenez N. (2018). Differential involvement of synovial adipokines in pain and physical function in female patients with knee osteoarthritis. A cross-sectional study. *Osteoarthritis and Cartilage*.

[41] Martel-Pelletier J., Raynauld J. P., Dorais M., Abram F., Pelletier J. P. (2016). The levels of the adipokines adipsin and leptin are associated with knee osteoarthritis progression as assessed by MRI and incidence of total knee replacement in symptomatic osteoarthritis patients: a post hoc analysis. *Rheumatology*.

[42] Gandhi R., Kapoor M., Mahomed N. N., Perruccio A. V. (2015). A comparison of obesity related adipokine concentrations in knee and shoulder osteoarthritis patients. *Obesity Research & Clinical Practice*.

[43] Lee J. H., Ort T., Ma K. (2009). Resistin is elevated following traumatic joint injury and causes matrix degradation and release of inflammatory cytokines from articular cartilage *in vitro*. *Osteoarthritis and Cartilage*.

[44] Wang K., Xu J., Cai J., Zheng S., Yang X., Ding C. (2016). Serum levels of resistin and interleukin-17 are associated with increased cartilage defects and bone marrow lesions in patients with knee osteoarthritis. *Modern Rheumatology*.

[45] Song Y. Z., Guan J., Wang H. J. (2016). Possible involvement of serum and synovial fluid resistin in knee osteoarthritis: cartilage damage, clinical, and radiological links. *Journal of Clinical Laboratory Analysis*.

[46] Toussirot E., Michel F., Bereau M. (2017). Serum adipokines, adipose tissue measurements and metabolic parameters in patients with advanced radiographic knee osteoarthritis. *Clinical Rheumatology*.

[47] Koskinen A., Vuolteenaho K., Moilanen T., Moilanen E. (2013). Resistin as a factor in osteoarthritis: synovial fluid resistin concentrations correlate positively with interleukin 6 and matrix metalloproteinases MMP-1 and MMP-3. *Scandinavian Journal of Rheumatology*.

[48] Poonpet T., Honsawek S. (2014). Adipokines: biomarkers for osteoarthritis?. *World Journal of Orthopedics*.

[49] Dong N., Gao Y. H., Liu B. (2018). Differential expression of adipokines in knee osteoarthritis patients with and without metabolic syndrome. *International Orthopaedics*.

[50] Yeung E. H., Sundaram R., Xie Y., Lawrence D. A. (2018). Newborn adipokines and early childhood growth. *Pediatric Obesity*.

[51] Lopes L. R., Ribeiro S. M. L. T., Figueiredo V. P. (2018). The overweight increases circulating inflammatory mediators commonly associated with obesity in young individuals. *Cytokine*.

[52] Degawa-Yamauchi M., Bovenkerk J. E., Juliar B. E. (2003). Serum resistin (FIZZ3) protein is increased in obese humans. *The Journal of Clinical Endocrinology and Metabolism*.

[53] Gross J. B., Guillaume C., Gegout-Pottie P., Mainard D., Presle N. (2014). Synovial fluid levels of adipokines in osteoarthritis: association with local factors of inflammation and cartilage maintenance. *Bio-medical Materials and Engineering*.

[54] Azamar-Llamas D., Hernandez-Molina G., Ramos-Avalos B., Furuzawa-Carballeda J. (2017). Adipokine contribution to the pathogenesis of osteoarthritis. *Mediators of Inflammation*.

[55] Massengale M., Lu B., Pan J. J., Katz J. N., Solomon D. H. (2012). Adipokine hormones and hand osteoarthritis: radiographic severity and pain. *PLoS One*.

[56] Fioravanti A., Cheleschi S., de Palma A. (2017). Can adipokines serum levels be used as biomarkers of hand osteoarthritis?. *Biomarkers*.

[57] DeClercq V., Cui Y., Forbes C. (2017). Adiposity measures and plasma adipokines in females with rheumatoid and osteoarthritis. *Mediators of Inflammation*.

[58] Patel L., Buckels A. C., Kinghorn I. J. (2003). Resistin is expressed in human macrophages and directly regulated by PPAR gamma activators. *Biochemical and Biophysical Research Communications*.

[59] Yin L., Wu Y., Yang Z. (2018). Characterization and application of size-sorted zonal chondrocytes for articular cartilage regeneration. *Biomaterials*.

[60] Zhang Z., Xing X., Hensley G. (2010). Resistin induces expression of pro-inflammatory cytokines and chemokines in human articular chondrocytes via transcription and mRNA stabilization. *Arthritis and Rheumatism*.

[61] Zhang Z., Zhang Z., Kang Y. (2014). Resistin stimulates expression of chemokine genes in chondrocytes via combinatorial regulation of C/EBP*β* and NF-*κ*B. *International Journal of Molecular Sciences*.

[62] Tarkowski A., Bjersing J., Shestakov A., Bokarewa M. I. (2010). Resistin competes with lipopolysaccharide for binding to toll-like receptor 4. *Journal of Cellular and Molecular Medicine*.

[63] Li Z., Wang X., Pan H. (2017). Resistin promotes CCL4 expression through toll-like receptor-4 and activation of the p38-MAPK and NF-*κ*B signaling pathways: implications for intervertebral disc degeneration. *Osteoarthritis and Cartilage*.

[64] Miao J., Benomar Y., al Rifai S. (2018). Resistin inhibits neuronal autophagy through Toll-like receptor 4. *Journal of Endocrinology*.

[65] Li B., Fang J., Zuo Z. (2018). Activation of the porcine alveolar macrophages via toll-like receptor 4/NF-*κ*B mediated pathway provides a mechanism of resistin leading to inflammation. *Cytokine*.

[66] Daquinag A. C., Zhang Y., Amaya-Manzanares F., Simmons P. J., Kolonin M. G. (2011). An isoform of decorin is a resistin receptor on the surface of adipose progenitor cells. *Cell Stem Cell*.

[67] Lee S., Lee H. C., Kwon Y. W. (2014). Adenylyl cyclase-associated protein 1 is a receptor for human resistin and mediates inflammatory actions of human monocytes. *Cell Metabolism*.

[68] Sanchez-Solana B., Laborda J., Baladron V. (2012). Mouse resistin modulates adipogenesis and glucose uptake in 3T3-L1 preadipocytes through the ROR1 receptor. *Molecular Endocrinology*.

[69] Wakeel A., Kuriakose J. A., McBride J. W. (2009). An *Ehrlichia chaffeensis* tandem repeat protein interacts with multiple host targets involved in cell signaling, transcriptional regulation, and vesicle trafficking. *Infection and Immunity*.

[70] Sato H., Muraoka S., Kusunoki N. (2017). Resistin upregulates chemokine production by fibroblast-like synoviocytes from patients with rheumatoid arthritis. *Arthritis Research & Therapy*.

[71] Zhang Z., Bryan J. L., DeLassus E., Chang L. W., Liao W., Sandell L. J. (2010). CCAAT/enhancer-binding protein *β* and NF-*κ*B mediate high level expression of chemokine genes CCL3 and CCL4 by human chondrocytes in response to IL-1*β*. *The Journal of Biological Chemistry*.

